# Observations on a Fistula in the Stomach, through Which the Interior of This Viscus Could Be Seen

**Published:** 1802-10-01

**Authors:** Le Roux


### *Observations on a Fistula in the Stomach, through which the interior of this Viscus could be seen; by* Cit. Corvissart *and* Le Roux.

A Woman happened to fall in a violent manner upon the threſhold of a door, when ſhe was in her twentieth year, by which the left ſide of the thorax and epigaſtric region were conſiderably hurt; ſhe recovered however after ſome time, and performed her uſual work, though there remained in that region a painful a painful ſenſation, which ſhe endeavoured to alleviate by bending the body towards the left, and by holding the hand on the affected ſide. In this ſtate ſhe had remained for about eighteen years, when an oblong tumour made its appearance at the painful place. Twenty days after an aperture was occaſioned in it, while the patient was vomiting, through which the water iſſued which ſhe had juſt ſwallowed. She felt herſelf, however, much relieved by it. She cloſed this fiſtula with a compreſs, through which a quantity of gaſtric humours continually oozed; and eight months after, the food began to paſs through the aperture, cauſing at the ſame time much pain. The margins of the wound became red, and the aperture gradually increaſed, and after three years its diameter was .0015 by .0010; it was ſituated at the anterior extremity of the eighth and ninth rib. Eight years after, this woman, at the age of forty-ſix, went to Paris in a carriage from a place forty-four leagues diſtant, without experiencing the leaſt inconvenience; ſhe was received in the Hoſpice de la Charité. The fiſtula had then an oval form, and its margins were of a red colour; the interior of the ſtomach could be diſtinctly ſeen to be furrowed with longitudinal folds, and covered with a gloſſy mucus. Whenever the patient introduced food into the ſtomach, it could be ſeen to deſcend at every deglutition, in form of a cylinder, attended with a certain quantity of air. Almoſt inſtantly, however, the food iſſued out from the opening by a kind of periſtaltic motion, which was produced by the tranſverſal plicae cloſing over one another.

Every day, about three or four hours after a meal, the patient let the food paſs out from the aperture, to which ſhe was ſtimulated by a particular diſagreeble ſenſation. Several attempts had been made to apply different ſtopples, but ſhe could bear none, except a double compreſs; ſhe uſed to waſh the ſtomach every evening with a pint and a half of water, after which ſhe went to bed and ſlept pretty well. She went to ſtool only once every ten days, and the excrements were hard, yellowiſh, and in ſmall quantity. Such was the ſtate of the patient, when experiments were firſt made with the matter that iſſued from the ſtomach. On examining chemically a frothy liquor, which was found every morning to be accumulated in the ſtomach, and which may be conſidered as the ſuccus gaſtricus, it was found to have the moſt ſtriking analogy with ſaliva. The food which ſhe had kept in the ſtomach for above three hours was alſo examined, and compared with that not yet conſumed; its weight had been conſiderably augmented by the addition of a certain quantity of gelatina, and of a ſubſtance which which had the greateſt analogy to the materia fibroſa of the blood. The proportion of muriat of ſoda and phoſphat of ſoda and of lime had been likewiſe increaſed. Theſe experiments were intended to be continued, when an acute diſeaſe carried her off within three days. On opening the body, the abdominal viſcera were found in a ſound ſtate; the ſtomach adhered to the ſides of the abdomen, and the fiſtula was ſituated on its anterior part, ſeven fingers breadth from the cardia and four from the pylorus.

